# Perceptions of patients and caregivers toward the management of rare disease in Malaysia: a qualitative research study

**DOI:** 10.1017/S0266462324000333

**Published:** 2024-10-24

**Authors:** Azuwana Supian, Asrul A. Shafie, Lock-Hock Ngu, Hatijah Ayob, Nathorn Chaiyakunapruk

**Affiliations:** 1 Pharmaceutical Services Programme, Hospital Tuanku Ja’afar Seremban, Ministry of Health Malaysia, Malaysia; 2 Discipline of Social and Administrative Pharmacy, School of Pharmaceutical Sciences, Universiti Sains Malaysia, Penang, Malaysia; 3 Genetic Clinic, Hospital Kuala Lumpur, Kuala Lumpur, Malaysia; 4 Malaysian Rare Disorders Society, Petaling Jaya, Malaysia; 5 Departments College of Pharmacy, University of Utah, Salt Lake City, UT, USA

**Keywords:** rare disease, orphan drug, Malaysia, access to medicine, patients’ perception

## Abstract

**Objective:**

The management of rare diseases is rarely addressed among policymakers and public communities. It is hindered by the lack of information on its epidemiology and burden, especially from the perspective of patients and families with rare diseases. This study aims to understand the perceptions of rare disease patients and their families in the management of rare diseases in Malaysia.

**Methodology:**

A qualitative interview was used to explore the perceptions of patients and families regarding the management of rare diseases in Malaysia. In-depth interviews were conducted with the rare disease patients or their parents/guardians provided by three major rare disease advocacy groups, between 1 July and 15 September 2016. The interviews focused on two key areas: the challenges associated with rare disease and the issues related to accessing medication.

**Findings:**

Out of the nineteen recruited participants, seventeen (89.5 percent) completed the interview sessions. The significance of awareness, knowledge, and support from others emerged as crucial for families and patients living with rare diseases. Despite facing delayed diagnosis and treatment, a majority of patients and parents expressed satisfaction with the advancements in rare disease management. Nevertheless, a prominent challenge revolves around access to enzyme replacement therapy for eligible patients.

**Conclusion:**

This study emphasizes the importance of healthcare professionals understanding patient with rare diseases perceptions to tailor communication strategies, provide accurate information, and address concerns effectively. The message underscores the significance of collaboration between healthcare providers and patient support groups to deliver adequate health information, potentially enhancing patients’ understanding and their illness perceptions.

Providing health care to all citizens is an essential service for any government. The competence of the health care system is a recurring and relevant key topic in health policy discussions. Everybody should be treated equally and with dignity. Rare diseases affect a small percentage of the population, are serious, and can be life-threatening (1). There are about 6,000 to 8,000 rare diseases and most of them are associated with genetic disorder that can be inherited or derived from gene mutations or chromosomal abnormalities. It was estimated that there are 400 million people worldwide currently suffering from rare diseases (2). According to the European Society of Paediatric Oncology, 75 percent of rare diseases affect children, of whom 30 percent die before reaching their fifth birthday (3). Research has shown that some rare diseases have a more harmful effect on health-related quality of life (QoL) than other serious diseases. However, it is always subject to the nature of individuals’ perceptions and factors beyond the influence of the physical manifestation of their QoL (4).

In reality, people with rare diseases are missing out on treatments when primary policy focuses on other health challenges such as communicable and non-communicable chronic diseases (5). The majority of them are undertreated because many healthcare professionals do not recognize the symptoms, or in certain cases, patients are not aware of the proper channels for treatment (6). The Department of Health UK, 2013 reported that four in every ten patients found it difficult to get a correct diagnosis (7). The difficulty in obtaining a correct diagnosis can frustrate patients who persistently seek a doctor capable of identifying their condition (8).

Despite the advancement of healthcare services in Malaysia, it still faces many shortcomings due to a lack of knowledge, awareness, and financial resources (9). Generally, the awareness level among Malaysian physicians about rare diseases is still low compared to their familiarity of other diseases. Overall, Malaysia has only five health centers with thirteen rare disease specialists and a dozen medical doctors who are trained in rare diseases (9). The management of rare disease is rarely addressed among policymakers and public communities. It is hindered by the lack of information on its epidemiology and burden, especially from the perspective of patients and families with rare diseases. By listening to them, we can understand the disease itself from their perspective: what are their experiences, barriers, and challenges? This study aims to understand the perceptions of rare disease patients and their families regarding the management of rare diseases in Malaysia.

## Methodology

In this study, a qualitative interview was used to explore patients and families’ perceptions toward the management of rare diseases in Malaysia. The interviews sought to better understand the challenges faced by individuals and families affected by rare diseases in accessing services and support, as well as to gauge the willingness to pay the unmet medical needs associated with rare disease treatment.

A formal letter requesting support and collaboration was sent to the three leading rare disease societies in Malaysia: the Malaysian Rare Disorders Society (MRDS), the Malaysia Lysosomal Diseases Association (MLDA), and the Malaysia Metabolic Society (MMS). The purpose was to seek their assistance in identifying patients with rare diseases and their families who were willing to participate in this interview. Open-ended questions were formulated to enable respondents to express their views and share experiences during the interviews.

Ethical approval was obtained from The Malaysia Medical Research & Ethics Committee (NMRR-14-1880-22900 (IIR)) and the Clinical Research Centre Hospital Kuala Lumpur (HCRC.IIR-2016-04-071).

Nineteen participants were identified through the societies, and interviews were scheduled between 1 July and 30 September 2016. Participants provided written informed consent to participate in this study and conducted face-to-face semi-structured interviews in English and Malay language for about 60 to 90 minutes (Supplementary Appendix 1). Each interview starts by inquiring related background and demographic information. The interviews focused on two sections as Table 1; (i) the issues and problem related to rare disease (diagnosis, information, and rare disease management in Malaysia), and (ii) the issues and related problem with access to the medicine (treatment, cost, and willingness to pay). Before the conclusion of the interview session, a summary of the interview notes will be reiterated for confirmation.

**Table 1.** Theme of topic guides

Issues and problem related with rare disease	
Theme 1	Experience in getting correct diagnosis
Theme 2	Experience in getting information on rare disease
Theme 3	Experience with health care system in rare disease management
Issues and problem related with access to the medicine	
Theme 4	Experience in getting treatment
Theme 5	Cost of treatment and willingness to pay
Theme 6	Expectation for rare disease health care system in Malaysia

The interviews were audio-recorded by the first author; and then transcribed verbatim by research assistants. The first author anonymized the transcribed text using unique codes, and the data were then entered into Atlas.ti (version 8) qualitative software. The analysis involved repeated readings of transcripts and identification of core themes and codes. Transcripts in Malay language were translated into English before inclusion in the results.

## Findings

Out of the nineteen individuals identified through the three societies, seventeen (89.5 percent) completed the interview sessions. One individual declined to participate, and efforts to contact another were unsuccessful. Table 2 provides an overview of the participants’ characteristics.

**Table 2.** Characteristics of the participants

Code RD-	Respondent (race)	Patient age (year)	Type of rare disease	Year of diagnosis/treatment	Treatment	Funding	Clinic visit/month	Distance from home
C1	Parent (M)	12	MPS II	2010/2015	Idursulfase (Elaprase)	Society (MLDA)	4	50–100 km
C2	Parent (M)	6	MPS I	2012/2014	Laronidase (Aldurazyme)		4	<50 km
C3	Parent (M)	6	MPS I	2011/2015	Laronidase (Aldurazyme)		4	<50 km
C4	Patient (M)	32	MERRF	2013/2013	Co–enzyme Q10 L–carnitine	MOH	Every 3 months	<50 km
C5	Parent (M)	5	MPS I	2013/2014	Laronidase (Aldurazyme)	MOH	4	50–100 km
C6	Parent (M)	13	Metabolic urea cyclic disorder	2003/2003	Special diet and milk	MOH	Eery 3 months	>100 km
C7	Patient (C)	26	MPS IVA	1992/2016	Elosulfase (Vimizim)	Society (MLDA)	4	<50 km
C8	Parent (M)	17	MPS II	2008/2011	Idursulfase (Elaprase)	MOH	4	<50 km
C9	Parent (I)	11	MPS VI	2008/2011	Galsulfase (Naglazyme)	MOH	4	>100 km
C10	Parent (C)	28	Pompe	2012/2015	Alglucosidase (Myozyme)	Society (MLDA)	2	>100 km
C11	Parent (M)	29	MPS VI	2011/2014	Galsulfase (Naglazyme)	MOH	4	>100 km
C12	Parent (I)	20	Fragile X	2004/NA	no treatment	NA	NA	NA
C13	Patient (M)	33	SMA	2006/NA	no treatment	NA	NA	NA
C14	Parent (M)	11	MPS II	2011/2014	Idursulfase (Elaprase)	ICAP	4	<50 km
C15	Parent (C)	16	MPS II	2006/2012	Idursulfase (Hunterase)	MOH	4	>100 km
C16	Parent (C)	7	MPS II	2011/2015	Idursulfase (Elaprase)		4	<50 km
C17	Parent (M)	3	MPS II	2013/2013	Idursulfase (Elaprase)	ICAP	4	>100 km

*Note:* Race: C, Chinese; I, Indian; M, Malay.

ICAP, International Compassionate Assistance Program; MERRF, Myoclonic epilepsy associated with ragged red fibers; MLDA, Malaysia Lysosomal Disease Association; MPS, Mucopolysaccharidosis; NA, not applicable.

Fifteen participants agreed to be interviewed face-to-face during their visit to the Genetic Clinic at Hospital Kuala Lumpur (HKL), while two interviews were conducted at the participants’ homes. During the 3-month interview period at HKL, the interviewer interacted with all patients in the Genetic Clinic. The majority of respondents were parents (82.3 percent) reflecting the fact that most patients were still children. Only three patients were able to actively participate and answer questions in the interview sessions. Almost two-third (70.6 percent) of the patients were diagnosed with mucopolysaccharidosis (MPS), a lysosomal storage disorder that necessitates the use of orphan drugs for treatment.

### Theme 1: Experience in Getting the Correct Diagnosis

Many patients received their rare disease diagnoses at a young age, typically during childhood. Lysosomal disorders, in particular, can often be detected in babies. However, due to doctors’ lack of knowledge and experience in rare diseases, patients faced delays in diagnosis or were misdiagnosed. Some patients only obtained an accurate diagnosis after a decade.

One participant shared, “*From birth, he was ok … However, when he was one year old, he had a fever on and off every month. At the age of 6, he was referred to a specialist in a tertiary hospital and diagnosed with a heart problem. The heart valve surgery was done, but we still did not know any other problems. Until he was 8 or 9 years old when we met geneticists… at last, we knew that he was suffering from MPS type II. It took almost ten years to know the actual illness*” (RD-C8).

At times, individuals outside the medical profession can notice unusual characteristics in these children. Teachers, for example, could observe abnormalities among their students and raise concerns to caregivers. Another participant shared, *“He was born normal… looks like other children. Until he was six years old, and he went to kindergarten. The teacher said that he had a dislike (feature, character) compared to other children. However, I ignore it and think nothing is serious. One day, my child had a fever, and I brought him to a public clinic. From there, the doctor told me that my son had a disorder and referred the case to the exper*t” (RD-C1).

In some cases, patients only confirmed their diagnosis in adulthood, often due to their relatively healthy state before reaching maturity or after a family member’s rare disease diagnosis. A participant expressed, *“I am an active and energetic man. Suddenly, I feel weak … it is strange. I took some supplements as I thought that maybe I was stressed, tired, and working too hard. I was able to learn about this genetic clinic from my uncle as he also experiences the same illness. So, all my family members did blood testing. And finally, my brother and I were confirmed diagnosed with the same disease … MERRF”* (RD-C4).

These interviews highlight a low level of awareness among physicians, healthcare providers, and general public. Rare diseases are infrequently encountered in clinics, resulting in misdiagnoses. Furthermore, the limited availability of laboratory testing, genetic, and metabolic clinics necessitates that patients and caregivers in other states travel considerable distances to Kuala Lumpur for testing services.

### Theme 2: Experience in Getting Information About the Rare Disease

The awareness, knowledge, and support from others are vital for those families and patients living with rare diseases. As anticipated in this study, none of the participants had prior knowledge of rare diseases before their personal experiences. Their introduction to rare diseases occurred when they or their family members were diagnosed with this illnesses that they had not heard from doctors, other people, or mass media before. Due to limited interaction time with healthcare professionals, participants turned to the internet to self-educate about rare diseases. Patient advocacy groups also play a significant role in providing information and support to these families and patients.

One participant emphasized the lack of awareness, stating, *“If it wasn’t because of me, all people around me like my parents, relatives, and friends would never know about this rare disease. I wouldn’t know about this condition either. I think the need to create more awareness on rare diseases is critical”* (RD-C7).

Another participant highlighted the overall low knowledge and awareness of rare diseases in most places, even within health clinics. They mentioned the absence of informational posters on rare diseases and the limited knowledge among healthcare providers, stating, *“I think the knowledge and awareness about rare disease are still low and none in most places. Although we go to a health clinic, there is no poster on rare disease information. The majority of healthcare providers also do not know much about this disease”* (RD-C12).

### Theme 3: Experience with Healthcare System in Rare Disease Management

The majority of patients received care and follow-ups from geneticists at HKL, the referral center for rare diseases in Malaysia. Participants, including both patients and parents, expressed overall satisfaction with the progress in rare disease management, essential treatments, and facilities provided at the genetic clinic. However, a significant issue revolved around the accessibility of enzyme replacement therapy (ERT) for eligible patients.

One participant noted, *“Overall, the health system in Malaysia is good. The only major problem I can see is a lack of experts and resources in this field (rare disease). My son has been waiting a few years for the ERT… and sadly, not all patients will get the medicines”* (RD-C2).

Another participant shared a positive perspective, stating, *“I think the health system in Malaysia, especially in rare disease management, is getting better. In previous years, the blood had to be sent to Australia for disease diagnosis confirmation. But now, I heard that it could be done locally in Malaysia”* (RD-C5).

### Theme 4: Experience in Getting Treatment and Access to the Medicines

In this study, nearly 60 percent of interviewed patients commenced treatment for their rare disease 3 years after a confirmed diagnosis. Meanwhile, two patients did not have specific medicine at the moment but received symptomatic treatment when necessary during follow-up clinics. Some patients secured treatment funding through crowdfunding, non-governmental organizations (NGOs), corporate bodies, and sponsorship from pharmaceutical industry programs, such as the International Compassionate Assistance Program (ICAP).

One participant shared their experience, stating, *“At first, I thought there is no medicine to treat this disease. My son was waiting for ERT’s medicine for quite some time (five years). No provision from the hospital allocation. Luckily, the MLDA sponsored the ERT… donations from the public”* (RD-C1).

For those sponsored by corporate bodies, the agreement was for a 6-month sponsorship, with the need to reapply for continuation subject to allocation. Another participant explained, *“My son was diagnosed with a rare disease when he was almost two years old. He only gets ERT when he reaches the age of four because his drug costs are costly. So, we waited more than two years before getting funds from a corporate company where my husband worked. However, it is a yearly basis, and I do not know next year whether it is funded or not”* (RD-C2).

ERT treatment were scheduled weekly at the genetic clinic, requiring patients to spent a day every week accordingly. Parents faced challenges such as risking job loss when taking leave to accompany their child weekly. They should be in the clinic early in the morning to monitor their child’s health status before receiving slow intravenous ERT for about 4 to 6 hours using an infusion pump.

One parent expressed the challenges, saying, *“My child is eligible for ERT after waiting for two years. However, we had to move here … thousands of kilometres from our hometown in Sabah as the treatment is scheduled once every week in HKL. I also need to talk to my boss and leave one day each week”* (RD-C2).

Half of the patients had to commute more than 100 km every week for ERT treatment at the genetic clinic in HKL. Families spent significant amounts on transportation and other expenses weekly and monthly. In other cases, three families decided to relocate from other states (Sabah, Pahang, Johore) when their kids were confirmed to receive ERT allocation at the genetic clinic in HKL.

A parent shared, *“My eldest son waited for three years to get this ERT. When treatment started, we had to travel 400 kilometres to HKL every week for three years. I spent a lot on transportation and food. Then with the help of the geneticist, I managed to rent a low-cost house in Kuala Lumpur”* (RD-C8).

### Theme 5: Cost of Treatment and Willingness to Pay

The treatment goals for patients with rare diseases encompass preventing the further accumulation of harmful substances, improving metabolic abnormalities, and eliminating toxic metabolites. It is important to note that not all rare diseases necessitate expensive medicines; some require standard medication, supplements, or special diets. However, patients with lysosomal disorder require ERT, incurring costs exceeding half a million every year. This amount poses a significant financial burden for patients, with some expressing their ability to afford only a few hundred to a few thousand for their treatment.

One participant highlighted financial constraints, stating, *“We are not rich people … we only can afford MYR300. Unfortunately, the cost of ERT is about a million every year … ordinary people will not be able to afford it”* (RD-C1).

Another participant expressed hope for government support, saying, *“It costs millions for a year. I don’t know for how many years I can survive, but I hope the government can subsidize it… maybe 60 percent; then I can work toward the remaining balance 40 percent by asking charity or fundraising”* (RD-C7).

Due to limited government funding for rare disease treatments, patients seek support from various sources, including pharmaceutical industries and NGOs. However, this assistance falls short of meeting the substantial and long-term financial needs of patients.

### Theme 6: Perception of Patients and Caregivers toward Rare Disease Management and Healthcare System in Malaysia

In general, the patients and caregivers express satisfaction with the Malaysian health system and rare disease management, noting improvements compared to previous years. However, there is a shared desire for these services to be more accessible, ideally closer to their homes, and to reach a level comparable to other countries, such as Taiwan, which has established a robust rare disease management system.

One participant stated, *“I was transferred here four years after my child received ERT treatment. The overall health system is good. However, rare disease services are needed and should be available in every state”* (RD-C3).

Another participant voiced their hope for a dedicated rare disease center in Malaysia, similar to Taiwan, stating, *“I hope there is a rare disease centre in Malaysia like in Taiwan. They have a rare disease act that will take care of rare disease management. The children can be diagnosed and get their ERT earlier, which will prevent their condition from deteriorating”* (RD-C7).

## Discussion

Illness perceptions, representing patients’ cognitive beliefs about their health conditions, significantly influence behavior during illness and correlate with various health-related outcomes (10). Positive illness perceptions are generally associated with better illness management, improved health outcomes, and informed healthcare-seeking behaviors (11). This study emphasizes that healthcare providers, despite being a prominent source of information among rare disease patients, are not necessarily linked to patients’ illness perception. Conversely, the positive influence of fellow patients is highlighted, revealing that individuals with acquaintances sharing the same diagnosis tend to have more positive illness perceptions across various aspects, they feel more in control, less worried, and understand better. Moreover, respondents relying on patient associations as information sources are more likely to feel that they can better comprehend their illness.

The results of this study align with findings from other research highlighting the lack of knowledge among doctors, which contributes to delays in diagnosing patients with rare diseases or leads to misdiagnoses (12;13). In the realm of rare diseases, the importance of early diagnosis is undeniable. In addition to physicians, paramedics – particularly nurses in public health clinics – play a pivotal role in identifying signs and symptoms in newborns and young children during routine health check-ups and vaccinations. Providing training for nurses to recognize indicators of rare diseases is imperative for facilitating early referrals and interventions.

Addressing the complexities associated with managing rare diseases requires an intensified focus, backed by both local and international efforts in awareness-building, research, and funding models. Collaborative endeavors involving patients, families, physicians, and advocacy groups can contribute significantly to raising public and political awareness regarding rare diseases. Furthermore, sharing information on a global scale can assist countries in adopting and adapting best practices, fostering partnerships and cooperation across different regions.

Helen Clark, the former administrator of the United Nations Development Programme, had stated in 2016 *“No country can claim to have achieved universal health coverage (UHC) if it has not adequately and equitably met the needs of those with rare diseases”* (14). This statement triggered many countries to implement the UHC to handle rare diseases more seriously. Funding for orphan drugs poses a significant challenge within the context of universal health coverage (UHC). Despite the high costs associated with orphan drugs, particularly ERT, their expense should not impede patients’ rights.

Scarce resources often lead to delayed treatment, creating an inequitable situation. Rare disease societies in Malaysia play an active role in bridging the accessibility gap through awareness campaigns and fundraisers. Donations are utilized not only for medical treatment costs but also cover equipment, and laboratory tests. Ensuring easy access to orphan drugs, particularly ERT, for unfunded patients is a critical need that requires ongoing support from all stakeholders. To reduce the gap in access to orphan drugs, all parties must clearly understand the rare disease management, structure, and national plan. A comprehensive rare disease national strategy and legislation or guidelines should be placed, including public and private funding efforts.

Overall, the data collected for this study primarily consists of cases seeking treatment at healthcare facilities. However, it is important to note that many patients may still remain undiagnosed or have been incorrectly diagnosed. Additionally, the participants in this study were primarily patients or caregivers undergoing treatment with orphan drugs. As a result, we did not capture the perspectives of patients or parents who have not yet received or are still awaiting such treatment, thereby limiting the breadth of information and viewpoints in this study.

To address this limitation, future research should aim to include the perspectives of patients who receive only symptomatic treatment. Furthermore, obtaining accurate prevalence data for rare diseases is essential for effective management. This would require conducting population studies over an extended period, with collaborative efforts and funding from both public and private healthcare providers. Such comprehensive studies would enable a better understanding of the prevalence of rare diseases and inform more targeted and effective management strategies.

## Conclusion

Understanding patients’ illness perceptions is crucial for healthcare professionals to tailor communication strategies, offer accurate information, and address concerns or misconceptions effectively. The correlation between health information seeking and illness perception highlights the need to carefully assess the real information needs of rare disease patients. The goal is to deliver health information that fosters positive illness perceptions.

This underscores a pivotal message for all healthcare providers. By capitalizing on their role as the primary source of information, healthcare providers can engage in collaborative efforts with patient associations. This partnership is instrumental in delivering comprehensive health information to rare disease patients, fostering a deeper understanding and positively influencing their illness perceptions.

## Supporting information

Supian et al. supplementary material Supian et al. supplementary material

## Supplementary material

The supplementary material for this article can be found at http://doi.org/10.1017/S0266462324000333.

## References

[1] The European Parliament and of The Council. Regulation (EC) No 141/2000 of The European Parliament and of The Council of 16 December 1999 on orphan medicinal products. 2000.

[2] Kaplan W, Wirtz V, Mante A, et al. Priority medicines for Europe and the world – 2013 update. Geneva: WHO Library; 2013.

[3] The European Society of Paediatric Oncology. Rare diseases. 2014. Available from: http://www.siope.eu/activities/european-advocacy/rare-diseases/.

[4] Cohen JS, Biesecker BB. Quality of life in rare genetic conditions: A systematic review of the literature. Am J Med Genet A. 2010;152A(5):1136–1156. doi:10.1002/ajmg.a.33380. 20425818 PMC3113481

[5] WHO. World Health Organization regional Office for South-East Asia, health situation in the South-East Asia region 2001–2007. Geneva: World Health Organization; 2008.

[6] Almalki ZS, Alahmari AK, Guo JJ, Kelton CM. Access to orphan drugs in the Middle East: Challenge and perspective. Intractable Rare Dis Res. 2012;1(4):139–143. 25343087 10.5582/irdr.2012.v1.4.139 PMC4204565

[7] Department of Health UK. The UK strategy for rare diseases. London: Department of Health; 2013. p. 40.

[8] Hunter P. Adopting an orphan. EMBO. 2005;6(6):504–507.

[9] Shafie AA, Supian A, Ahmad Hassali MA, et al. Rare disease in Malaysia: Challenges and solutions. PLoS One. 2020;15(4):e0230850. 32240232 10.1371/journal.pone.0230850 PMC7117672

[10] Petrie KJ, Jago LA, Devcich DA. The role of illness perceptions in patients with medical conditions. Curr Opin Psychiatry. 2007;20(2):163–167. 17278916 10.1097/YCO.0b013e328014a871

[11] Lopes MT, Koch VH, Sarrubbi-Junior V, Gallo PR, Carneiro-Sampaio M. Difficulties in the diagnosis and treatment of rare diseases according to the perceptions of patients, relatives and health care professionals. Clinics. 2018;73:e68. 29641803 10.6061/clinics/2018/e68 PMC5866403

[12] Uhlenbusch N, Löwe B, Depping MK. Perceived burden in dealing with different rare diseases: A qualitative focus group study. BMJ Open. 2019;9:e033353. 10.1136/bmjopen-2019-033353 PMC6937088 31888936

[13] der Lippe C, Diesen PS, Feragen KB. Living with a rare disorder: A systematic review of the qualitative literature. Mol Genet Genom Med. 2017;5(6):758–773. 10.1002/mgg3.315 PMC5702559 29178638

[14] United Nations Development Programme. Helen Clark: Written statement at the 11th annual international conference on rare diseases and orphan drugs. 2016. Available from: http://www.undp.org/content/undp/en/home/presscenter/speeches/2016/10/20/helen-clark-written-statement-at-the-11th-annual-international-conference-on-rare-diseases-and-orphan-drugs.html.

