# Supplementary material for: Perceptions of patients and caregivers toward the management of rare disease in Malaysia: a qualitative research study
Source: Int J Technol Assess Health Care. 2024 Oct 24;40(1):e34. doi: 10.1017/S0266462324000333 (PMC11569895; doi:10.1017/S0266462324000333)
Supplement: Supian et al. supplementary material [file S0266462324000333sup001.docx]

Appendix 1

ID No:

Semi-structured interview guide for

**PATIENTS & FAMILIES**

**DEVELOPMENT OF ORPHAN DRUGS COVERAGE DECISION FRAMEWORK IN MALAYSIA**

*My name is* ***Azuwana Supian*** *and I am a PhD candidate of Universiti Sains Malaysia. My interests in this research are the issues of rare disease and orphan drug in Malaysia. How we can ensure adequate, continuous and equitable access to quality, safety, effectively and affordable medicines towards achieving optimal health outcomes. Maybe you can say or suggest something that can help the policy makers to consider new system or guideline of orphan drug in Malaysia.*

*I’m really interested in hearing what you have to say about this issue.*

*To begin with, could you please tell me something about yourself?*

| **Demographic data** | | | |
| --- | --- | --- | --- |
| Age | |  | Gender: M F |
| Status | | Patient Family | |
| Occupation | |  | Salary/income: RM ………………….. / month |
| Rare diseases | | ……………………………………… Diagnosed date: ………………………… | |
| Orphan drug(s) | | ……………………………………… Initiated date: ……………………………. | |
| **Section 1:** **Issues & problems** | | | |
| 1. | How have your experiences with the health care system in rare disease treatment? | | |
| 2. | Would you please describe to me the main problems of health care system in rare disease? | | |
| 3. | How have you experienced in getting diagnose of your disease?  Could you tell or describe the problems? | | |
| 4. | How have you experienced in getting information of your disease? | | |
| **Section 2:** **Access to the medicine and willingness to pay** | | | |
| 1. | How have you experienced in getting treatment of rare disease?  Could you tell or describe the problems of orphan drugs? | | |
| 2. | How are these problems affecting your daily life and overall quality of life?  (the impact on general activities of daily living, work, leisure, life aspirations, social life, mental health) | | |
| 1. | What do you think about public and major charitable funding bodies?  Is it enough funding available for rare disease treatment? | | |
| 2. | Until now, how much do you pay for the treatment and other expenses?  How much do you willing to pay for cost or co-payment of rare disease treatment? | | |
| 3. | What do you hope to achieve in healthcare system in Malaysia for rare disease patients? | | |

***Thank you for your time and participation!***
